# Vocal learning as a preadaptation for the evolution of human beat perception and synchronization

**DOI:** 10.1098/rstb.2020.0326

**Published:** 2021-10-11

**Authors:** Aniruddh D. Patel

**Affiliations:** ^1^ Department of Psychology, Tufts University, Medford, MA, USA; ^2^ Program in Brain, Mind, and Consciousness, Canadian Institute for Advanced Research, Toronto, Canada

**Keywords:** vocal learning, synchrony, rhythm, evolution, beat, gene-culture coevolution

## Abstract

The human capacity to synchronize movements to an auditory beat is central to musical behaviour and to debates over the evolution of human musicality. Have humans evolved any neural specializations for music processing, or does music rely entirely on brain circuits that evolved for other reasons? The vocal learning and rhythmic synchronization hypothesis proposes that our ability to move in time with an auditory beat in a precise, predictive and tempo-flexible manner originated in the neural circuitry for complex vocal learning. In the 15 years since the hypothesis was proposed a variety of studies have supported it. However, one study has provided a significant challenge to the hypothesis. Furthermore, it is increasingly clear that vocal learning is not a binary trait animals have or lack, but varies more continuously across species. In the light of these developments and of recent progress in the neurobiology of beat processing and of vocal learning, the current paper revises the vocal learning hypothesis. It argues that an advanced form of vocal learning acts as a preadaptation for sporadic beat perception and synchronization (BPS), providing intrinsic rewards for predicting the temporal structure of complex acoustic sequences. It further proposes that in humans, mechanisms of gene-culture coevolution transformed this preadaptation into a genuine neural adaptation for sustained BPS. The larger significance of this proposal is that it outlines a hypothesis of cognitive gene-culture coevolution which makes testable predictions for neuroscience, cross-species studies and genetics.

This article is part of the theme issue ‘Synchrony and rhythm interaction: from the brain to behavioural ecology’.

## 1. Introduction

Recent years have seen growing research on the evolutionary foundations of human musicality, defined as the spontaneously developing cognitive and sensorimotor abilities supporting musical behaviour [1]. Among these abilities is the capacity to synchronize rhythmic movements to an auditory beat. This ‘beat perception and synchronization’ (BPS) is a form of entrainment, whereby periodic motor and auditory rhythms become temporally coordinated with the close temporal alignment of movements to auditory beats. BPS is a prominent aspect of human musical behaviour, is not part of everyday spoken language and is foundational to dance [2]. Because BPS is culturally widespread, emerges without explicit instruction, begins developing early in life and builds on infant predispositions to move rhythmically to beat-based rhythms [3–9], it is relevant to evolutionary questions about musicality. Does BPS reflect any evolved neural specializations for music processing, or does it rest entirely on brain circuits that evolved for other reasons? This question goes to the heart of a long-standing debate about evolution and music. In *The Descent of Man* (1871), Darwin [10] argued humans had evolved to be musical, while in *The Principles of Psychology* (1890), William James [11], who admired Darwin and believed the human mind was full of instincts, implied that music was a purely cultural invention built on brain mechanisms that evolved to serve other functions. Nearly 150 years later, we are nowhere near consensus on this debate, with detailed arguments on both sides (e.g. [12–14] versus [15–17]). BPS is important to this debate because it is the focus of a growing body of research relevant to evolutionary questions, spanning neuroscience, cross-species studies, genetics, cross-cultural work and developmental psychology (e.g. [18–21]).

Thanks to research in cognitive neuroscience, several key behavioural and neural features of BPS are well known. One such feature is the spontaneous tendency of listeners to predict the timing of beats, as shown by experiments where people tap to the beat of auditory rhythms: taps fall within a few tens of milliseconds of beats and often anticipate the beat, indicating precise temporal prediction of beat times [22]. Another key feature is tempo flexibility. When engaging in BPS humans have a preference for beats separated by approximately 500 ms, near a natural frequency of human movement [23]. However, humans exhibit precise and predictive temporal synchronization (e.g. taps closely aligned to beats) across a wide range of tempi, spanning inter-beat-intervals ranging from approximately 250 ms to approximately 1 s [24]. Precise temporal prediction and tempo flexibility enable tight coordination in group music-making and dance. Thus, these abilities are probably important to the social functions and psychological consequences of synchronized, collective musical behaviour [25–27].

In terms of neuroscience, BPS can be separated into perceptual and motor processes, which do not always co-occur because listeners can perceive a beat while remaining still (although see [28–30]). Yet brain imaging has revealed that even in the absence of movement, beat perception and motor system activity are linked. When humans perceive a beat in an auditory rhythm, several motor areas, including cortical premotor and subcortical motor control areas, are strongly active and interacting (e.g. [31–34]). Overall, the networks involved in beat perception without movement and in BPS have a great deal of overlap [35,36], and there is growing interest in the idea that motor system activity plays a causal role in predicting the timing of beats even when humans do not move to the beat [37–41].

According to one line of theorizing and computational modelling, BPS taps into ancient and widespread brain mechanisms of entrainment. These mechanisms involve endogenous cortical neural oscillations which become coupled to stimulus-driven oscillations of brain activity caused by sensory input [42,43]. This view reflects an old intuition that musical rhythm processing is rooted in fundamental aspects of animal biology. Darwin voiced this intuition in *The Descent of Man* (1871), when he wrote ‘The perception, if not the enjoyment, of musical cadences and of rhythm is probably common to all animals, and no doubt depends on the common physiological nature of their nervous systems' [10, vol. 2, p. 333]. One appeal of the coupled oscillator framework is that it has been productively applied to diverse forms of biological entrainment, ranging from circadian rhythms to synchrony in insect choruses [44,45], and is thus attractive for its generality. Indeed, commenting on his model of adaptive coupled oscillators for the synchronization of rhythmic firefly flashing in tropical trees, Ermentrout [45, p. 584] noted ‘In a broader context, the equations we consider in this paper are similar to those describing an interconnected oscillatory neural network… The only significant difference between the firefly tree and an oscillatory neural network is in the time scale (milliseconds for the neural network and seconds for the firefly tree) and the space scale (microns for the neural system and meters for the insects).’ Another reason for the appeal of a coupled oscillator framework for BPS is research suggesting that neural oscillations have an important role to play in sensory, motor and cognitive processing across a range of species, including humans [46–48].

The coupled oscillator perspective on BPS entails the idea that many animal species should be capable of this form of rhythmic entrainment, since the purported brain mechanisms are very general. Yet BPS is notably absent in our most familiar animal companions, such as, dogs, cats and farm animals, even though these species have lived with humans and their music for thousands of years [49,50]. Wilson & Cook [51] have suggested that this does not reflect the lack of a capacity for BPS and is owing to other factors that inhibit BPS from developing spontaneously. These could include a lack of motivation for BPS, inattention to auditory rhythms because of their ecological irrelevance for the animal, or the fact that the rhythms of human music are not at tempi suited to the natural frequencies of the animal's movements. Consistent with this view, Cook and colleagues showed that a California sea lion (*Zalophus californianus*), which did not spontaneously engage in BPS when exposed to rhythmic music, could learn to synchronize her head bobs to a musical beat via operant training [52].

A very different hypothesis suggests that the capacity for BPS is limited to a narrow range of species. The ‘vocal learning and rhythmic synchronization hypothesis' proposed that BPS relies on specialized auditory–motor forebrain circuitry which originally evolved to serve complex vocal learning [53]. In complex vocal learning, an animal requires auditory input to develop its normal species-specific vocalizations, because this input forms an auditory template which guides the development of the animal's own vocalizations [54]. Complex vocal learning occurs in a few groups of mammals, including cetaceans, pinnipeds and humans uniquely among primates, and in three groups of birds: songbirds, parrots and hummingbirds.

The vocal learning hypothesis (VLH) predicts that only species with complex vocal learning are capable of BPS. This motivated a number of studies testing the hypothesis, including research demonstrating BPS in parrots, which supported it [55,56]. However, the sea lion study mentioned above challenged the hypothesis, because sea lions do not show evidence of complex vocal learning. While there are open questions about whether the sea lion study refutes the VLH, as discussed in §3 below, there are other reasons to revisit the hypothesis. One such reason is the growing view that vocal learning should not be considered a dichotomous trait that animals have or lack, but a more continuous trait along which animals vary, or a modular trait with distinct subcomponents which can dissociate in different species [57–60]. Another reason to revisit the hypothesis is that parrots appear to be the only nonhuman vocal learners to show spontaneous BPS to human music,^1^ despite the fact that songbirds have complex vocal learning and are sometimes extensively exposed to human music as pets [56,63]. This is important because recent neural research has revealed that the parrot vocal learning system is more elaborate than that of songbirds [64]. A final reason to revisit the VLH is the considerable amount of neurobiological research on beat processing and vocal learning which has taken place in the 15 years since the hypothesis was published.

Collectively, these factors suggest it is time to reconsider the hypothesis that complex vocal learning is related to the emergence of BPS in our species. The current paper addresses this goal and is organized as follows: I first clarify the scope, background and evolutionary implications of the original VLH. I next critically examine the data that challenge the hypothesis and then discuss the implications of a continuum/modular view of vocal learning for the hypothesis. Based on these considerations, I propose a revision of the hypothesis whereby an advanced form of vocal learning acted as a preadaptation for BPS. I then suggest that once this form of vocal learning evolved in our lineage, it interacted with ancestral primate rhythmic vocal behaviours to produce sporadic BPS as a fortuitous trait, i.e. intermittent BPS in response to periodic auditory rhythms. Finally, I propose that our capacity for BPS was sharpened by gene-culture coevolution, leading to evolved neural specializations for sustained BPS in humans. I describe the predictions this view makes in terms of neuroscience, cross-species studies and genetics, and close by discussing the larger significance of research on BPS for theoretical issues in evolutionary biology.

## 2. The original vocal learning and rhythmic synchronization hypothesis

In this section, I discuss the scope, background and evolutionary implications of the original vocal learning and rhythmic synchronization hypothesis [53] (henceforth, VLH). Understanding the scope of the VLH is particularly important, because it explains why the hypothesis has not been challenged to date by any studies other than the sea lion research mentioned above.

### (a) Scope

The VLH focuses on BPS, which differs in several ways from well-known cases of rhythmic entrainment in other species, as noted in earlier publications [53,65]. First, BPS includes the capacity to align rhythmic movement to a periodicity perceived in complex auditory rhythms, distinguishing it from synchronization to quasi-metronomic auditory patterns as seen in crickets and katydids [66–68]. Second, BPS involves predictive and temporally precise alignment of movement with beats over a wide tempo range (e.g. from approximately 50% slower to 100% faster than one beat every 500 ms, as noted previously), contrasting with the narrower ranges of tempi over which some insects can synchronize their periodic sonic pulses in a phase-aligned fashion [69–71]. Third, BPS often involves movements which are not themselves aimed at sound production, such as head bobbing or rhythmic movements of the arms or trunk [72], unlike rhythmic entrainment in insect acoustic chorusing, which is aimed at sound production.

To date, research on synchronization of movement to an auditory beat in monkeys has focused on metronomes (e.g. [73,74]) or tempo-varying metronomes [75], and thus does not challenge the VLH, as a defining feature of BPS is the ability to synchronize to periodicities perceived in complex auditory rhythms. Nevertheless, such research has been a valuable source of neural data on how primate brains coordinate rhythmic movements with rhythmic sounds, and these data have informed neural theories of human beat perception [41]. Interestingly, primate research suggests that monkeys and humans have similar capacities in terms of single-interval or ‘absolute’ timing, but differ in capacities related to beat-based or ‘relative’ timing, supporting the gradual audiomotor evolution hypothesis [76]. This hypothesis is consistent with neural research on rhythm perception in monkeys which finds that they are sensitive to isochrony but do not appear to perceive a beat in complex rhythms [77,78].

In terms of predictive and tempo-flexible synchronization with a beat, a striking finding from monkey research is that when trained to synchronize movements to a metronome, their spontaneous tendency is to move reactively rather than predictively with respect to the stimulus, unlike humans [73,79]. Recent research has shown monkeys can be trained to synchronize predictively if every predictive movement is rewarded [75,80], raising the idea that species may lie along a continuum of ability or proclivity for reactive versus predictive motor synchronization with a beat. Interestingly, while rats appear to share monkeys' spontaneous tendency for reactive motor synchronization to metronomes [81], Hattori *et al*. [82] demonstrated that a chimpanzee showed spontaneous predictive synchronization of movement to a metronome. However, this only occurred at one tempo near the animal's spontaneous tapping tempo, and the animal showed no evidence of tempo flexibility. Research with a bonobo has shown that it occasionally synchronized predictively during concurrent rhythmic drumming with a human partner [83], but because the bonobo and human could see each other it is unclear if the animal is capable of BPS without support from visual rhythmic signals. This is a concern as research with other primates reveals that they are better at synchronizing movement with discretely timed visual versus auditory rhythms, unlike humans [22,75]. Finally, despite anecdotal reports of spontaneous BPS in horses trotting to music without a rider onboard who might cue them to the beat, empirical work has not provided evidence of tempo flexibility in this behaviour, although methods for testing this have been developed and research is currently underway [84,85].

### (b) Background and evidence

When the VLH was proposed in 2006, there were no known cases of BPS in nonhuman animals, either spontaneous or trained, and it had been suggested that BPS was uniquely human [86]. The VLH was motivated by a synthesis of behavioural, neural and cross-species research. Behavioural experiments had shown that humans were far better at extracting a beat and synchronizing to it in complex auditory versus visual rhythms matched in temporal structure [22]. (Later work extended this auditory advantage to matched complex tactile rhythms [87,88].) This suggested that beat perception involved specialized auditory–motor processing. When considering evolutionary forces which might have strengthened auditory–motor processing in humans, complex vocal learning was a plausible candidate as it involves tight coupling between auditory input and motor output in order to match vocal movements to an auditory model [89,90]. In terms of neural work, brain imaging had revealed that even in the absence of movement, beat perception engages a number of motor regions of the human brain [31,91], including premotor and basal ganglia (striatal) regions. Interestingly, it was known from research on birds that the evolution of complex vocal learning was associated with neural specializations in premotor and striatal regions [90]. Collectively, these findings led to the VLH, which proposed that the neural circuitry for complex vocal learning was a necessary prerequisite for the capacity for BPS [53].

The key prediction of the VLH hypothesis was that vocal non-learning animals would not be capable of BPS, while only animals with complex vocal learning would have this capacity. (It is worth noting that while the VLH claimed the neural circuitry for complex vocal learning was a *necessary* foundation for BPS, it never claimed it was *sufficient* [65].) Research demonstrating BPS to music in large parrots, such as the sulfur-crested cockatoo ‘Snowball’, supported the hypothesis [55,56] and experimental work on pecking to a metronome in small parrots (budgerigars) also proved consistent with the hypothesis [92], though synchronization to a beat in more complex auditory stimuli remains to be studied in smaller parrots.

### (c) Evolutionary implications

The VLH suggested that brains shaped by evolution for complex vocal learning had ‘BPS potential’ as a byproduct of their wiring. As parrots are not known to engage in BPS as part of their natural behaviour [93], the discovery of BPS in these animals supported the theoretical position that key components of human musicality rely on brain circuits that evolved for other reasons, and that humans have not evolved neural specializations for music processing [2,11,15–17,94].

## 3. A critical examination of data challenging the vocal learning hypothesis

To date, the only challenge to the VLH is from a study of a California sea lion (‘Ronan’) who was trained to synchronize her head bobs to a musical beat and who showed tempo flexibility in generalizing this ability to novel, untrained tempi ranging from 20% slower to 10% faster than the original tempo of 130 beats per minute (BPM) [52]. Because sea lions are not known to be complex vocal learners, this excellent study challenges the VLH. Yet several facts suggest that this study does not refute the VLH.

First, while sea lions are traditionally considered a vocally inflexible species, the upper limits of their vocal learning capacities are not yet known [95] and have not been studied using modern methods applied to other pinnipeds [96]. This is a concern because there is strong evidence of complex vocal learning in pinnipeds such as grey seals and harbour seals [96,97]. Indeed, it is already known that adult sea lions are more vocally flexible than macaque monkeys, as the former, but not the latter, can be trained to vocalize on command [95]. (Interestingly, juvenile macaques (*Macaca mulatta*) can be trained to vocalize on cue, but unlike sea lions this ability is lost as they grow into adulthood, even as they retain the ability to make non-vocal movements on command [98,99].) Given sea lions' voluntary control of vocalization, it would be interesting to see if they can be trained to modulate spectral or temporal aspects of their calls, which would indicate even greater vocal flexibility.

Second, Ronan was tested as a juvenile, between 3 and 4 years of age, before the age of sexual maturity in this species, which is around 4–5. Juveniles in some mammalian and avian species are more vocally flexible than adults [100,101]. Furthermore, related to a point made by Schachner [102], the intensive operant training Ronan received in synchronizing movements to sounds (first with metronomes, later with music), combined with the heightened plasticity of juvenile mammalian brains [103], raises the question of whether Ronan developed unusually strong auditory–motor forebrain circuitry compared to normal sea lions. This could be addressed via structural brain imaging of auditory–motor forebrain connections in Ronan compared to conspecifics without early auditory–motor training, using recently developed *in vivo* magnetic resonance imaging (MRI) protocols for sea lions [104].

Third, as noted by Merker *et al*. [105], the way Ronan synchronized her movements to music was unlike human BPS in an important respect. Specifically, there was a strong change in the phase relationship of her head bobs to the musical beat as a function of tempo. When tested for her ability to synchronize at novel tempi, Ronan's head bobs lagged considerably behind the beat by an average of approximately 90^o^ at the fastest tempo (143 BPM) and occurred considerably before the beat by an average of approximately 60^o^ at the slowest tempo (117 BPM) (fig. 5 of [52]). This is unlike human BPS, in which rhythmic movements and beats remain much more phase aligned across a comparable range of tempo variation. Ronan's pattern of phase leads and lags is reminiscent of an oscillator with a single intrinsic period driven by nearby frequencies [106]. Subsequent elegant work with Ronan studying her synchronization to metronomes with phase or tempo perturbations [107], and modelling her behaviour with an oscillator capable of period and phase correction, showed that Ronan exhibited a low degree of period coupling to the stimulus, below the range of period coupling reported in prior human studies. This suggests her synchronization abilities may reflect different mechanisms from those used by humans, perhaps relying more heavily on subcortical circuits.

Based on the above points, I believe that rejecting the VLH on the basis of Ronan's rhythmic entrainment abilities is premature. Nevertheless, the pioneering studies of Cook, Rouse and colleagues [52,107] are an important challenge to the VLH and motivate further work on pinnipeds' rhythmic synchronization abilities. As a clade with a broad range of vocal flexibility across species [95], pinnipeds are a particularly promising group for studying phylogenetic relationships between vocal learning and rhythmic synchronization in mammals [108,109].

## 4. Vocal learning as a continuous or modular trait

The original VLH proposed that complex vocal learning provided the evolutionary and neural foundations for BPS. As noted above, complex vocal learners require auditory input to develop their normal species-specific vocalizations, because this input acts as a template that guides vocal development [54]. Complex vocal learning is associated with specializations of auditory–motor forebrain circuitry which support sophisticated auditory–motor neural interactions [110]. This type of vocal learning can be distinguished from limited vocal learning, where the latter is ‘the ability to fine-tune acoustic features of species-specific vocalizations that can develop in the absence of auditory input because innate motor programs can generate the species-specific pattern’ [54, p. 3]. While humans are the only primate with complex vocal learning, limited vocal learning occurs more broadly in primates, including in chimpanzees [111,112]).

The distinction between complex and limited vocal learning highlights the fact that vocal learning is not a binary trait that animals have or lack. Indeed, this has been known for some time. Songbird researchers have long distinguished between closed-ended and open-ended complex vocal learners, where the former, such as zebra finches, do not modify their repertoire after the initial song learning, while the latter, such as European starlings, continue to modify their repertoire as adults. Furthermore, complex vocal learners can differ in other ways, including in whether they copy a tutor model or improvise on tutor songs, and whether they ‘copy only tutor songs that fit tightly constrained species-specific parameters or copy essentially anything they hear’ [113].

**Figure 1.**
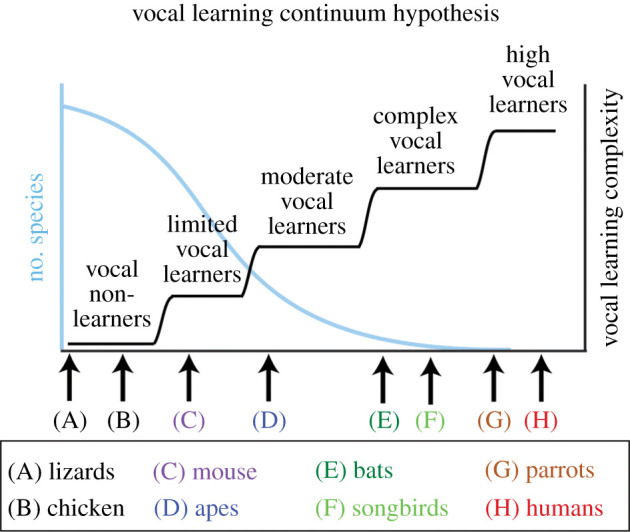
The vocal learning continuum hypothesis, from Petkov & Jarvis [57], updated by Jarvis [60]. Diagram of hypothesized stepwise continuous ability of vocal learning among vertebrates (right *y*-axis), from simple to more complex forms (*x*-axis). As vocal learning complexity increases, there are a decreasing number of species with the ability (left *y*-axis). (A–H) Proposed example species at each step on the continuum. The continuum ranges from lizards that do not vocalize and have no vocal learning, to nonhuman primates with limited vocal learning, to songbirds with complex vocal learning, to parrots and humans with high vocal learning. (Figure and caption modified from [60] with permission from the author.) (Online version in colour.)

Of direct relevance to the VLH, recent theoretical and empirical work suggests a continuum in vocal learning capacities across species, or regards vocal learning as comprised multiple distinct abilities that may be targeted independently by evolutionary pressures [57–59]. These include the ability to flexibly coordinate the timing of innate vocalizations in social interactions with conspecifics, such as in the antiphonal calling seen in marmosets and singing mice [114,115], and the ability to modify vocalizations as a function of social and auditory experience, or ‘vocal plasticity’. Vocal plasticity is the most relevant for the VLH, because substantial modification of the spectral and temporal properties of vocalizations based on auditory experience requires extensive auditory–motor crosstalk in the forebrain, and extensive, precise auditory–motor cortical crosstalk is a foundation of BPS. In this regard, the ‘vocal learning continuum hypothesis' [57,58,60] is of particular interest, as it suggests that vocal plasticity varies in a quasi-continuous way across vertebrates. As shown in figure 1, the hypothesis groups parrots and humans together as high vocal learners at a far end of this continuum, with greater vocal plasticity than complex vocal learners.

**Figure 2.**
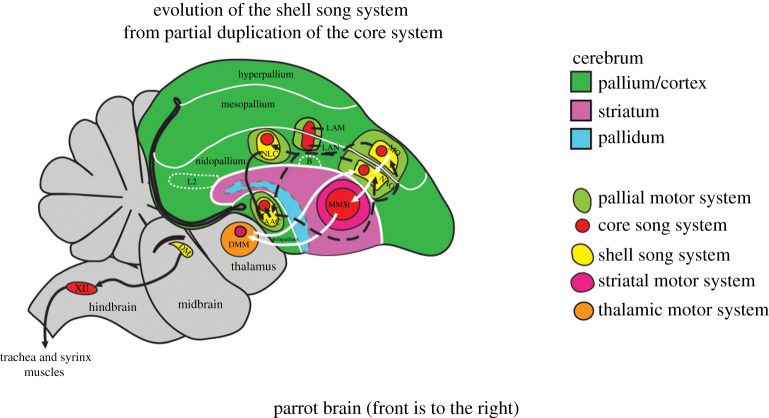
Schematic of the vocal system in a parrot brain, adapted from Chakraborty & Jarvis [116] with permission from the authors and The Royal Society. Red regions, core song system (similar to songbirds); yellow regions in pallium/cortex, shell song system (unique to parrots). The shell system is proposed to have evolved out of a partial duplication of the core song system. Black solid arrows, posterior vocal motor pathway; white solid arrows, anterior vocal motor pathway; dashed arrows, connections between core and shell systems. Not all connections are shown for simplicity. See caption of the original figure in [116] for definitions of acronyms. (Online version in colour.)

This placement makes intuitive sense because it has long been known that parrots have extraordinary vocal plasticity, being able to imitate human speech and other sounds with high fidelity. In support of placing them in a distinct category relative to other complex vocal learners, recent neural research using gene expression and neural tract tracing [64,116] has revealed that parrots have a uniquely elaborated vocal learning system, consisting of a ‘core’ system similar to that found in songbirds, and a ‘shell’ system unique to parrots (figure 2).

While the relative contributions of the core and shell systems to parrot vocal plasticity remain to be understood, one intriguing possibility is that these distinct yet interconnected systems facilitate independent control of the syrinx (the avian analogue of the mammalian larynx) and tongue during vocalization. It has been found that parrots modulate both of these anatomical structures to shape vocal sounds [117], whereas in songbirds the acoustic structure of songs is largely produced by the syrinx rather than the tongue [110]. Regardless of the precise functional significance of the elaborated vocal control system of parrots, Chakraborty & Jarvis [116] propose that it enhances vocal–auditory–motor integration compared to the songbird system and suggest that this is related to parrots' abilities to synchronize body movements to musical rhythms.^2^

**Figure 3.**
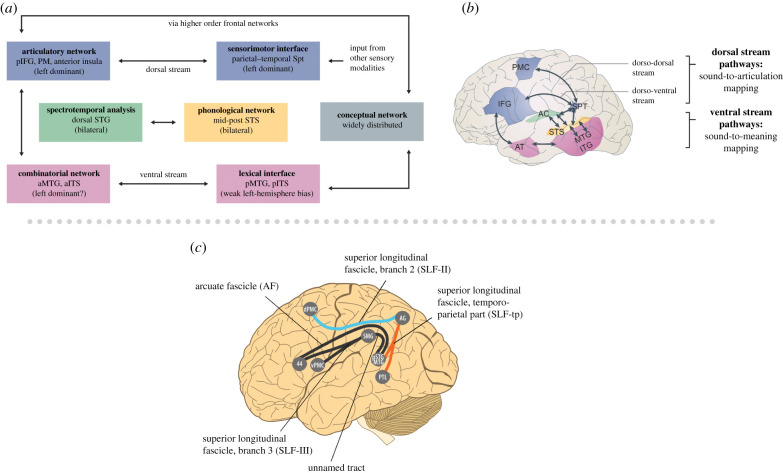
(*a,b*) Dual stream model of spoken language processing, adapted from Hickok & Poeppel [119]. Colours in the functional modules of (*a*) are matched to brain regions in (*b*), which shows neural pathways with dashed lines. Acronyms in (*b*): PMC, premotor cortex; IFG, inferior frontal gyrus; SPT, sylvian parieto-temporal area; AC, auditory cortex; STS, superior temporal sulcus; MTG, middle temporal gyrus; ITG, inferior temporal gyrus AT, anterior temporal cortex. (*c*) A more detailed view of dorsal stream pathways involved in spoken language (from [50], adapted from [122]). Of particular interest for BPS are connections shown in orange and blue: orange connections link secondary auditory regions in the posterior superior temporal gyrus/middle temporal gyrus (pSTG/MTG) and parietal regions near the angular gyrus (AG), and blue connections link regions near the angular gyrus to the dorsal premotor cortex (dPMC). These connections correspond to two branches of the superior longitudinal fasciculus (SLF): the temporo-parietal branch (SLF-tp) and the second branch (SLF II). Both tracts appear to play a role in sound-to-articulation mapping, which is part of vocal learning, and have been proposed to support auditory–motor interactions serving beat perception [38]. Other acronyms in (*c*): PTL, posterior temporal lobe; SMG, supramarginal gyrus; vPMC, ventral premotor cortex; 44, Brodmann area 44 (part of Broca's area). (Online version in colour.)

The discovery of a dual pathway system for vocal learning and control in parrots is fascinating in light of an influential model of human speech processing in which the sensorimotor control of speech relies on two pathways within a complex ‘dorsal stream’ which bidirectionally connects cortical premotor and auditory regions via the temporo-parietal cortex [119]. These two pathways are probably important to our ability to independently control the larynx and tongue in shaping vocal sounds [120,121]. Figure 3*a*,*b* shows a schematic of this model, which distinguishes dorsal and ventral streams in spoken language processing, and which has two pathways within the dorsal stream, labelled dorso-dorsal and dorsoventral. In this model, dorsal stream pathways are involved in speech sound-to-articulation mapping and ventral stream pathways are involved in mapping perceived sounds onto lexical representations. Figure 3*b* shows a more elaborate diagram of dorsal stream pathways based on a review of long-distance neural connections involved in auditory language processing [122].

Relevant to the VLH, neural studies in humans indicate the involvement of dorsal stream regions in BPS and in beat perception without overt movement, including the dorsal premotor cortex and parietal cortex, which are connected by the blue pathway in figure 3*c* [32,34,35,123]. Patel & Iversen [38] proposed that dorsal stream pathways are crucial for communicating temporal predictions about beat timing from premotor to secondary auditory regions, via the parietal cortex. Based on prior neuroanatomical research in monkeys [124], Patel and Iversen further proposed that a specific part of the dorsal stream is much more strongly developed in humans than in monkeys owing to the evolution of vocal learning in our lineage, namely the orange fibre tract in figure 3*c* linking auditory regions in posterior superior temporal gyrus to regions around the angular gyrus. The relative weakness of this connection in monkeys could help explain why they do not spontaneously move predictively when synchronizing to auditory rhythms.

Stepping back to the larger picture, the additional neural regions and pathways for vocal learning in parrots compared to complex vocal learning songbirds are intriguing when juxtaposed with the complexity of the human dorsal auditory stream, which is involved in vocal learning and BPS. This juxtaposition, combined with the fact that only parrots and humans show spontaneous BPS to music, suggest that spontaneous BPS has an evolutionary relationship to high vocal learning rather than to complex vocal learning more generally.

## 5. The revised vocal learning and rhythmic synchronization hypothesis

The original VLH proposed that the capacity for BPS relied on neural substrates that first evolved to serve complex vocal learning. Based on the considerations reviewed above, here I propose the revised vocal learning and rhythmic synchronization hypothesis or ‘rVLH’. Like the VLH, the rVLH is focused on BPS, which differs in several ways from synchronous rhythmic behaviours seen in insects and many other species, as previously discussed. However, unlike the original VLH, the rVLH shifts the focus from complex vocal learning as a prerequisite for BPS. Rather, it seeks to explain why spontaneous BPS occurs in ‘high vocal learners’ such as humans and parrots, who have a behaviourally and neurally more elaborate form of vocal learning than complex vocal learners (figure 1 and §4 above). The rVLH proposes that high vocal learning is a preadaptation for spontaneous, sporadic BPS to periodic auditory rhythms. This is because:

(i) high vocal learning provides intrinsic rewards for predicting the temporal structure of complex auditory sequences, because such predictions scaffold vocal learning of such sequences;
(ii) temporal predictions about auditory periodicities in the hundreds-of-milliseconds range are made via action-like neural processes in forebrain motor planning regions; and
(iii) in high vocal learners, these motor planning regions are in tight reciprocal communication with forebrain auditory regions throughout life.

The first reason above is based on cognitive research on statistical learning, a form of implicit learning without external reinforcement which involves detecting patterns and regularities in the environment [125]. Research suggests that statistical learning of auditory sequences is an active process in which predictions are continuously formed and compared to incoming input in order to update mental models of sequence structure [125,126]. Santolin & Saffran [127] point out that statistical learning of auditory patterns by humans begins in infancy as part of language acquisition and also occurs in nonhuman animals. Notably, they argue that ‘statistical learning is likely to drive vocal learning in organisms that must learn to produce structured vocalizations’ (p. 59). These authors also discuss research indicating that parrots surpass songbirds in the ability to learn underlying patterns in sound sequences (see also [128]). Point (i) above asserts that part of the cognitive system of high vocal learners is a mechanism that provides them with an intrinsic reward for accurately predicting the temporal structure of such sequences, to facilitate learning of this structure. This relates to an idea raised later in this paper, namely that selection on the motivation for BPS can be conceptually distinguished from the selection on the capacity for BPS.

The second reason in the above list is based on recent theorizing about the role of the motor system in predicting timing in periodic auditory rhythms [41]. This theorizing draws heavily on neurophysiological research on rhythmic timing in nonhuman primates (e.g. [74,129,130]). The involvement of the motor system in such predictions, combined with the above idea of intrinsic rewards for such predictions, provides an explanation for spontaneous rhythmic movement to auditory rhythms in high vocal learners.

The third reason in the above list is based on the importance of strong reciprocal connections between forebrain premotor and auditory regions for high vocal learning and for BPS. Circuit-level research on the role of such connections in vocal learning has only been conducted in songbirds, who have complex vocal learning [131]. Because high vocal learners surpass complex vocal learners in vocal flexibility and in the neural complexity of their vocal learning system (cf. §4), the strength and plasticity of such auditory–motor interactions is probably stronger in high vocal learners than in complex vocal learners. The rVLH argues that these strong connections allow rapid two-way communication between forebrain auditory and motor planning regions, scaffolding the spontaneous predictive movements to auditory rhythms seen in high vocal learners.

The rVLH's claim that high vocal learning is a preadaptation for spontaneous, sporadic BPS to periodic auditory rhythms requires clarification of some terms. The first is ‘preadaptation’, which is ‘an evolutionary change that adapts organisms to one set of environmental conditions but in addition and quite fortuitously positions them for a new surge in adaptive evolution’ [132, p. 13]. Examples of preadaptation abound in evolutionary biology, as evidenced by research in palaeontology, developmental biology and genetics [133]. Feathers, for example, were a preadaptation for flight, evolving in therapod dinosaurs long before flight evolved in their avian descendants [134]. Feathers originally served non flight-related functions such as thermal insulation, conferring flight-related aerodynamic benefits as a fortuitous consequence of their structure. Only later were the flight-related properties of feathers a direct target of natural selection, resulting in changes in feather structure supporting powered flight [135]. Just as the aerodynamic properties of early feathers were a fortuitous byproduct of their structure, the rVLH suggests that a capacity for sporadic BPS was a fortuitous byproduct of the neural circuitry for high vocal learning.

A second term requiring clarification is ‘sporadic.’ Sporadic BPS is the type of BPS observed in parrots, whereby rhythmic movements are phase aligned to an auditory beat during sporadic ‘bouts’ of several seconds surrounded by stretches of little movement or of rhythmic movement not synchronized to the beat. This is what was observed in Snowball, who exhibited BPS in bouts with a median of 16 head bobs, and tended to gravitate to a head bob tempo near 126 BPM during unsynchronized movement to music [55,136]. When presented with 10 different novel tempi relative to the original musical tempo of 109 BPM, Snowball synchronized in a phase-aligned manner at nine of these tempi spanning 98–130 BPM [55]. While statistical analyses showed that this amount of synchronization was very unlikely to happen by chance, Snowball's sporadic BPS is distinct from the sustained BPS observed in adult humans, where rhythmic movements remain phase aligned to a beat for much longer periods, even in musically untrained individuals [72]. Interestingly, sporadic BPS may be more representative of how young children move to music [9,137].

The third term that requires clarification is ‘spontaneous’, meaning BPS that emerges without explicit instruction or physical rewards, as observed in humans and in parrots. Snowball the cockatoo, for example, was never explicitly trained to move rhythmically to music using food rewards, unlike the sea lion Ronan. Of course, parrots, like human children, often receive positive attention from human adults for BPS, and such attention is doubtless rewarding in species that form strong and lasting social bonds, as parrots and humans both do [138]. Indeed, such social rewards may amplify BPS behaviour. However, by focusing on *intrinsic* rewards, the rVLH entails the idea that attention and social rewards alone cannot account for spontaneous BPS to music. This distinguishes it from a proposal made by Wilson & Cook [51, p. 1655], who suggest that parrots engage in BPS because ‘these birds bond with their caretakers and are highly sensitive to social reward, making it particularly likely that they will pick-up behaviours that humans find amusing’. A problem with this proposal is that dogs bond strongly with their carers and are highly sensitive to social reward [139], yet do not show spontaneous BPS to music [56,118].

A focus on the intrinsic rewards of temporal prediction in sequence processing bears a resemblance to a suggestion by Merker [140] that vocal learners have an intrinsic motivation for high-fidelity copying of sounds, because such copying is needed for vocal learning and is typically not reinforced by immediate external rewards. Merker refers to this motivational mechanism as a ‘conformal motive’ and suggests that in parrots this motivation to copy could extend to non-vocal body movements, leading them to imitate humans moving to the beat of music [105]. Consistent with this view, parrots can imitate non-vocal movements [141]. However, an intrinsic motivation to imitate non-vocal movements also seems to occur in chimpanzees. For example, a juvenile chimpanzee observing an adult crack a nut with an anvil stone and pounding stone will imitate the adult's actions without any reinforcement and often without success [142]. Yet despite this intrinsic motivation to imitate movements, among the cases of enculturated chimpanzees raised by humans and exposed to music during their development, there are no reports of spontaneous BPS in these animals [118].

Before closing this section, it is worth emphasizing that while the rVLH claims that the neural circuity for high vocal learning is a *necessary* prerequisite for spontaneous BPS, it does not claim that it is *sufficient*. To date, parrots and humans are the only species known to engage in BPS spontaneously. In addition to high vocal learning, parrots and humans also share the ability to imitate non-vocal movements and a tendency to live in complex social groups and form long-term bonds [65,118]. The extent to which these factors also act as preadaptations for spontaneous BPS merits further research [143].

## 6. The primate heritage in the origins of human beat perception and synchronization

The rVLH suggests that a brain adapted for high vocal learning will fortuitously show a predisposition for spontaneous, sporadic BPS. Yet for BPS to occur, exposure to rhythmic sound is needed. In the case of parrots living with humans, human music provides such rhythms. Assuming human ancestors had evolved high vocal learning, what would be the source of rhythmic sounds in their environment? Research on chimpanzees and bonobos, the closest living relatives of humans, shows that rhythmic vocalizations are part of their natural social behaviour. These vocalizations include pant hooting in chimpanzees and high-hooting in bonobos, and both species show evidence of temporal coordination with conspecifics when making these vocalizations [144–146]. Furthermore, short episodes of rhythmic drumming on tree buttresses are part of chimpanzee display behaviour in the wild [147], and an untrained chimpanzee in captivity has been filmed drumming steadily on a barrel for more than 30 s [148]. Thus, it seems plausible that rhythmically structured sounds produced in social contexts were present in the last common ancestor of humans and chimpanzees/bonobos [149]. The rVLH suggests that such sounds, in the context of a human ancestor that was a high vocal learner, could have led to spontaneous, sporadic BPS.

In light of this suggestion, a recent study by Hattori & Tomonaga [150,151] is of particular interest. These researchers found that enculturated chimpanzees exposed to complex rhythms made rhythmic rocking and swaying movements. These movements were not entrained to the beat and occurred whether the rhythms were beat-based or not, thus differing from BPS in important ways. Nevertheless, the movements seemed to reflect positive engagement, were made without any reinforcement and did not resemble distress responses or stereotyped behaviours sometimes seen in poorly treated animals. Furthermore, despite the fact that the chimpanzees were free to leave the testing area at any time, one male chimpanzee stayed closer to the sound source when the sounds were on versus off, suggesting attraction to the stimulus. Furthermore, this chimpanzee also made a few different types of rhythmic movements in response to rhythmic sounds, including head bobbing and hand clapping. When combined with field observations of chimpanzee ‘rain dancing’ (ritualized movements in response to loud sounds such as rain or waterfalls [152]), this study suggests that a predisposition to move rhythmically to loud, complex sound patterns may have been in place in human ancestors prior to the evolution of high vocal learning. In the framework of the rVLH, this would facilitate the occurrence of sporadic BPS once high vocal learning evolved.

## 7. Human beat perception and synchronization and gene-culture coevolution

There is growing evidence that gene-culture coevolution has shaped some important human biological traits. Convincing cases coming from biological adaptations to diet, including the evolution of lactose tolerance around 10 000 years ago in certain populations that practiced dairying, and much more ancient and species-wide anatomical changes associated with the control of fire and associated dietary changes [153–155]. In this section, I suggest that BPS has been the locus of cognitive gene-culture coevolution, with a gradual transition from sporadic to sustained BPS in human ancestors leading to evolved neural specializations for sustained BPS in humans. (Recall that in sporadic BPS, as observed in parrots, accurate synchronization to a beat occurs in short bouts of a few seconds separated by stretches of little movement or of unsynchronized rhythmic movement. In sustained BPS, accurate synchronization is maintained over longer stretches of time, as seen in a dance around the world today.) The current proposal aligns with modern theories suggesting a prominent role for gene-culture coevolution in the emergence of human musicality (e.g. [13,156–159]).

The previous section argued that sporadic BPS arose fortuitously when the neural circuitry for high vocal learning evolved in a human ancestor that already had rhythmic social vocalizations. Below I suggest why a transition from sporadic to sustained BPS occurred via gene-culture coevolution, and then outline predictions of this view in terms of neuroscience, cross-species studies and genetics. I close this section by briefly discussing two important issues for future work in this area.

### (a) Factors leading to gene-culture coevolution for beat perception and synchronization

For BPS to become a target of gene-culture coevolution, early humans would first have to employ sporadic BPS in a cultural behaviour that became widespread. Here, I would like to draw an analogy to the early use of fire use in human cultures. Fire use has a long history in the genus *Homo*, dating back at least 1.5 million years [160] and is widely believed to have led to changes in human biology via gene-culture coevolution, including anatomical and physiological adaptations to eating cooked food [154,161]. Current research suggests that fire use began with early humans taking advantage of ‘fortuitous fire’, such as from lightning strikes, sometimes transporting it to safe places such as caves [160]. The larger point is that fire use probably became widespread in human culture long before the ability to make fire at will and long before fire-driven gene-culture coevolution. This illustrates Richersen *et al.*'s [162, p. 8985] point that ‘Culture normally evolves more rapidly than genes, creating novel environments that expose genes to new selective pressures’.

In the case of fire, the spread of fire use based on scavenging fire from natural sources is easy to understand owing to controlled fire's use, e.g. in keeping warm and fending off predators. It is less clear why early humans would use and spread behaviours based on sporadic BPS. If sporadic BPS first emerged in the context of rhythmic social vocalizations in an ancestor with high vocal learning, as suggested in §6 above, perhaps early humans used sporadic BPS for social purposes. For example, Mehr *et al*. [14] have suggested that synchronized rhythmic vocalizations and body movements could act as signals of coalition strength aimed at other groups, and Merker *et al*. [163] suggest synchronized rhythmic vocalizations could have a ‘beacon-like’ effect, attracting potential mates and deterring rivals from a distance. If sporadic BPS supported such behaviours and the behaviours led to advantages in survival or reproduction, then displays using sporadic BPS could have spread through human groups via purely cultural dynamics, e.g. via imitation of more successful groups.

Yet if this occurred, why would gene-culture coevolution favour a transition to sustained BPS? Fisher & Ridley [164, p. 930] have suggested that ‘The smallest, most trivial new habit adopted by a hominid species could—if advantageous—have led to the selection of genomic variations that sharpened that habit’. A possible advantage of increasingly sustained (versus sporadic) BPS in social contexts is that it scaffolds sustained interpersonal synchrony, which can in turn influence subsequent social behaviour [165]. A large body of empirical work finds that group members engaging in sustained interpersonal synchrony are subsequently more cooperative and feel more socially connected to each other [166–168], and may also show more in-group conformity and obedience [169]. This may partly reflect the blurring of self-other boundaries owing to the way sustained interpersonal synchrony interacts with neural mechanisms of action, perception and prediction in the brain [27,170]. If these social effects were more pronounced when episodes of interpersonal synchrony were more sustained, and facilitated behaviours outside of musical contexts that promoted survival, then gene-culture coevolution could favour genetic variants enhancing the capacity and proclivity for sustained BPS. Here, ‘capacity’ refers to the neural mechanisms supporting sustained BPS, while ‘proclivity’ refers to neural mechanisms that make sustained BPS rewarding, especially in social contexts. (Selection on the proclivity for BPS, independent of selection on capacity, has been suggested previously [61].) With these ideas in mind, I turn to the predictions that emerge from this proposal.

### (b) Predictions of the hypothesis: neuroscience, cross-species studies and genetics

Several predictions emerge from the hypothesis that our current capacity for BPS is a product of gene-culture coevolution which refined a preadaptation for BPS rooted in high vocal learning. Because this hypothesis combines the revised rVLH with the claim of subsequent gene-culture coevolution, I will refer to it as the *rVLH*, using italics to distinguish it from the rVLH as outlined in §5 above, which makes no claim for gene-culture coevolution. Importantly, the predictions listed below are not made by the view that human rhythmic synchronization to a beat reflects widespread entrainment mechanisms with no special relation to the auditory–motor neural specializations of high vocal learners.

#### (i) Human neuroscience

**Figure 4.**
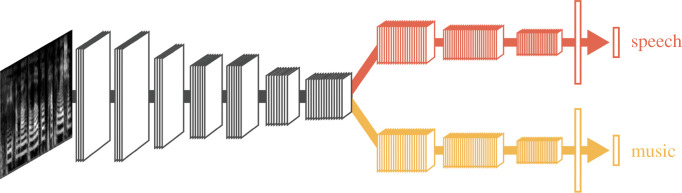
An optimized hierarchical artificial neural network for recognition of sounds as speech or music, after Kell *et al*. [172]. Auditory input is shown at the left (spectrogram-like representation of sound). Lower level processing stages shared by speech and music are shown in black and white, higher level stages and streams unique to each domain are shown in colour. (Online version in colour.)

The *rVLH* predicts significant overlap in the neural circuitry of BPS and high vocal learning in regions and pathways of the auditory dorsal stream, because BPS is seen as an evolutionary offshoot of high vocal learning. Such specialization could occur via evolutionary mechanisms of brain area duplication or brain pathway duplication/elaboration [116,171]. Note that ‘overlap’ does not mean ‘identity’. Owing to evolved neural specializations, the *rVLH* suggests that there should be some neural populations or pathways which show selective involvement in BPS versus vocal learning. One way to conceptualize this combination of sharing and specialization is suggested by recent computational modelling work on the neural relationship between music and speech processing. In a study in which hierarchical artificial neural networks were optimized for speech and music recognition [172], the best-performing networks showed substantial shared processing at early stages, followed by separate speech and music regions and pathways at higher stages (figure 4). This organization is consistent with neural research suggesting that music and speech processing have significant overlap in subcortical and primary auditory cortical regions [173–175] yet also have neurally specialized processing in non-primary auditory cortex [176–178].

The *rVLH's* prediction of neural overlap between BPS and high vocal learning is consistent with studies finding links between childhood speech-related abilities and non-linguistic beat processing [179–181]. At the same time, the fact that the *rVLH* posits evolved neural specializations for BPS is consistent with the existence of congenital ‘beat deafness’, in which individuals with normal hearing and musical pitch perception have severe problems perceiving and/or synchronizing with a musical beat [182,183].

The *rVLH* further predicts that human developmental neuroscience will reveal experience-expectant plasticity in circuits underlying the capacity for sustained BPS [184,185]. Finally, as noted at the end of the previous subsection, the *rVLH* suggests that natural selection acted not only on the capacity for sustained BPS, but also the proclivity or motivation to engage in this behaviour. The hypothesis thus calls for research on the relationship between BPS and reward-related activity in the brain. In particular, the *rVLH* suggests that the neural rewards humans experience when engaging in BPS-based interpersonal synchrony [186] may reflect evolved neural specializations in the striatum, which is involved both in beat perception and reward [41].

#### (ii) Cross-species studies

The *rVLH* predicts that the more advanced a species' vocal learning capacities (figure 1), the more its behavioural capacity for, and neural mechanisms of, BPS will resemble those of humans. Thus for example, it predicts that grey seals, which have recently been shown to have remarkable vocal mimicry abilities [96] will outperform less vocally flexible pinnipeds in studies of BPS. Similarly, it predicts that cetaceans with advanced vocal learning abilities, such as belugas and bottlenose dolphins [187,188], will outperform less vocally flexible cetaceans on tests of BPS. Counterintuitively, the *rVLH* predicts the brain mechanisms of rhythmic synchronization to a beat are more similar in humans and parrots than in humans and sea lions, owing to the convergently evolved similarities of parrots and humans in vocal learning capacities, and despite the much closer phylogenetic proximity of humans and sea lions.

In terms of neuroanatomy, because the *rVLH* posits that the advanced degree of auditory integration with motor circuits seen in high vocal learners is critical for spontaneous BPS and also posits that the auditory dorsal stream is a key site of this enhanced integration, it makes predictions for research on primate comparative neuroanatomy. Specifically, it predicts significant differences in auditory dorsal stream pathways important for BPS in humans and homologous pathways in other primates owing to the limited vocal learning abilities of those species. For example, the *rVLH* predicts that connections between the secondary auditory cortex and parietal cortex (red pathway linking superior temporal gyrus and parietal cortex in figure 3*c*, see §5) will be much stronger in humans than in monkeys or chimpanzees. This prediction can be tested using structural MRI methods such as diffusion tensor imaging, which are currently used to compare human, monkey and chimpanzee cortical connectivity [189,190].

#### (iii) Genetics

For BPS to be subject to gene-culture coevolution, it must have a heritable genetic substrate. Recently, a large-scale genome-wide association study with over 600 000 participants found 67 genetic loci associated with BPS, indicating a heritable, polygenic genetic substrate [20]. That is, BPS is a complex or ‘polygenic’ trait, in which interactions between genetic variants at different loci probably play an important role, rather than a ‘Mendelian’ trait influenced by variation at a single gene. The results of this study were virtually unchanged by controlling for more general tests of cognition, consistent with results from the twin literature which show that the genetics of rhythm are not solely attributable to general cognitive effects. Importantly, in this new study, genetic variance explained only about 13–16% of phenotypic variance in the beat synchronization trait, indicating that variance in BPS ability is genetically influenced but far from genetically determined.

The *rVLH* makes three distinct predictions about the genetic substrate of BPS. First, like the original VLH, it predicts genetic relationships between BPS and vocal learning. Supporting this prediction, new research [191] finds that the genetic architecture of BPS is significantly enriched for genes expressed during song production in songbird Area X, a key nucleus for vocal learning in avian brains, homologous to human basal ganglia. This is of interest as the basal ganglia plays an important role in human beat perception [33,41]. Second, since the *rVLH* proposes there was natural selection for sustained BPS after sporadic BPS emerged as a preadaptation, it predicts BPS will show independent genetic variation in humans after variance related to vocal learning abilities is accounted for. Third, the *rVLH* predicts that quantitative signatures of natural selection in the genome [192] will be found in the genetic substrate of BPS. In this regard, it is interesting that two loci associated with BPS in the study of Niarchou *et al*. [20] are in ‘human accelerated regions’, i.e. regions of the genome that are strikingly different from many other species, including our closest living primate relatives [193].

As a theory of gene-culture coevolution, the *rVLH* also motivates research on existing small-scale cultures which have traditionally had a very little collective, synchronized music-making [194]. In cases where such cultures have had limited gene flow with other groups, the rVLH predicts that individuals in those cultures will show signatures of relaxed selection on genetic variants supporting sustained BPS.

### (c) Two issues for future research

A key issue for future research on BPS and gene-culture coevolution is a more complete spelling out of the proposed feedback cycles between culture and genes in the evolution of BPS. For example, relationships between collective musical synchrony, behaviour outside of musical contexts, cultural group selection^3^ and individual-level selection remain to specified in detail. One goal of such theorizing is generating testable predictions distinct from those made by ‘classical’ evolutionary theories of musicality not involving interactions between cultural innovation and biological evolution.

Another issue for future work is broadening the lens when considering possible evolved neural specializations for BPS. The current paper has focused on forebrain circuitry as a site of such specializations, including cortical auditory–motor connections and motivation/reward circuitry in the striatum. Yet when humans engage in BPS, these forebrain circuits interact with lower brain regions, creating loops that include the midbrain, cerebellum, brainstem and spinal cord. Evolutionary changes relevant to BPS may not be restricted the forebrain, and future work will profit from an examination of BPS-relevant regions and connections throughout these loops [29,30,195].

## 8. Conclusion

This paper has reviewed and revised the hypothesis that the evolutionary foundations of human BPS lie in our capacity for complex vocal learning. On the basis of behavioural and neural research on vocal learning and on beat processing, the paper argues that an advanced form of vocal learning, convergently evolved in humans and parrots, acts as a preadaptation for sporadic BPS. It further argues that in humans, mechanisms of gene-culture coevolution transformed this preadaptation into a genuine neural adaptation for sustained BPS. This larger significance of this proposal for evolutionary biology is that it outlines a scenario of *cognitive* gene-culture coevolution which makes testable predictions for neuroscience, cross-species research and genetics. To date, convincing examples of gene-culture coevolution in humans come from studies of non-neural physiology or anatomy, e.g. related to digestion and diet. While there is great interest and promise in the idea that gene-culture coevolution has shaped human mental abilities, including language [155,162,164,196–198], we currently lack compelling biological evidence for cognitive gene-culture coevolution. The study of BPS has the potential to provide such evidence and could thus pave the way for other studies examining how biology and culture intertwined in the evolution of the human mind.

## Endnotes

1 Elephants in sanctuaries have been reported to synchronize rhythmic movements to an auditory beat [56], but as noted by the lead author of that study, it is not clear if this behaviour was spontaneous or trained, and tempo flexibility was not tested [61]. Elephants are vocal learners, but their exact degree of vocal flexibility is not yet known [62].

2 Interestingly, there do not appear to be major differences in the vocal learning circuitry of male and female parrots [64], and both male and female parrots have been found to synchronize rhythmic movements to a musical beat [118].

3 Note that the revised vocal learning hypothesis is not based on biological group selection theory.

## Data accessibility

This article has no additional data.
